# Cryoballoon pulmonary vein isolation as first line treatment for typical atrial flutter (CRAFT): study protocol for a randomised controlled trial

**DOI:** 10.1007/s10840-020-00746-6

**Published:** 2020-05-08

**Authors:** Wern Yew Ding, Emmanuel Williams, Moloy Das, Lilith Tovmassian, Muzahir Tayebjee, Guy Haywood, Claire Martin, Kim Rajappan, Matthew Bates, Ian Peter Temple, Tobias Reichlin, Zhong Chen, Richard Balasubramaniam, Christina Ronayne, Nichola Clarkson, Saagar Mahida, Christian Sticherling, Dhiraj Gupta

**Affiliations:** 1 Liverpool Heart and Chest Hospital NHS Foundation Trust, Thomas Drive, Liverpool, L14 3PE UK; 2 Institute of Ageing and Chronic Disease, University of Liverpool, Liverpool, UK; 3 Liverpool Centre for Cardiovascular Science, Liverpool, UK; 4 Newcastle Upon Tyne Hospital NHS Foundation Trust, Freeman Hospital, Newcastle, UK; 5 Leeds Teaching Hospital NHS Foundation Trust, Leeds, UK; 6 University Hospitals Plymouth NHS Foundation Trust, Plymouth, UK; 7 Royal Papworth Hospital NHS Foundation Trust, Cambridge, UK; 8 Oxford University Hospitals NHS Foundation Trust, Oxford, UK; 9 South Tees Hospitals NHS Foundation Trust, James Cook University Hospital, Middlesbrough, UK; 10 Manchester University NHS Foundation Trust, Wythenshawe Hospital, Manchester, UK; 11 Inselspital, Bern University Hospital, University of Bern, Bern, Switzerland; 12 Ashford and St Peter’s Hospital NHS Foundation Trust, Surrey, UK; 13 Royal Bournemouth and Christchurch Hospital NHS Foundation Trust, Bournemouth, UK; 14 University Hospital Basel, University of Basel, Basel, Switzerland

**Keywords:** Atrial flutter, Cavo-tricuspid isthmus, Cryoballoon, Radiofrequency, Ablation, Atrial fibrillation

## Abstract

**Purpose:**

Treatment of typical atrial flutter (AFL) with cavo-tricuspid isthmus (CTI) ablation is associated with a high occurrence rate of new onset atrial fibrillation (AF) during follow-up. There are data to support the addition of pulmonary vein isolation (PVI) to CTI ablation in patients with both AF and AFL, but the role of cryoballoon PVI only, with no CTI ablation, in AFL patients with no prior documentation of AF has not been studied.

**Methods:**

CRAFT is an international, prospective, randomised, open with blinded assessment, multicentre superiority study comparing radiofrequency CTI ablation and cryoballoon PVI in patients with typical AFL. Participants with typical AFL are randomised in a 1:1 ratio to either treatment arm, with patients randomised to PVI not receiving CTI ablation. Post-procedural cardiac monitoring is performed using an implantable loop recorder. The primary endpoint is time to first recurrence of sustained symptomatic atrial arrhythmia. Key secondary endpoints include (1) total arrhythmia burden at 12 months, (2) time to first episode of AF lasting ≥ 2 min, (3) time to recurrence of AFL or AT and (4) procedural and fluoroscopy times. The primary safety endpoint is the composite of death, stroke/transient ischaemic attack, cardiac tamponade requiring drainage, atrio-oesophageal fistula, requirement for a permanent pacemaker, serious vascular complications requiring intervention or delaying discharge and persistent phrenic nerve palsy lasting > 24 h.

**Conclusion:**

This study compares the outcomes of 2 different approaches to typical AFL—the conventional ‘substrate’-based strategy of radiofrequency CTI ablation versus a novel ‘trigger’-based strategy of cryoballoon PVI.

**Trial registration:**

(ClinicalTrials.gov ID: **NCT03401099**)

**Electronic supplementary material:**

The online version of this article (10.1007/s10840-020-00746-6) contains supplementary material, which is available to authorized users.

## Background

Typical atrial flutter (AFL) is a macro-reentrant tachycardia with a circuit within the right atrium involving the cavo-tricuspid isthmus (CTI) as the critical isthmus. In patients with AFL, the use of anti-arrhythmic drugs (AADs) to maintain sinus rhythm has limited success [1]. Radiofrequency ablation (RFA) of the CTI has a very high acute success rate and is often used as first line treatment. However, as many as half of these patients go on to develop atrial fibrillation (AF) during follow-up [2, 3]. The elevated risk of AF among patients with AFL indicates the presence of shared underlying disease processes that remain unchanged with only CTI ablation. In fact, there is evidence to suggest that pulmonary vein (PV) ectopy is a common initiating trigger for both arrhythmias [4, 5].

Previous studies have demonstrated the benefit of a combined pulmonary vein isolation (PVI) and CTI ablation approach among patients with AFL [6–8]. A small single centre study even showed the efficacy of standalone PVI in patients with typical AFL compared with AADs or CTI ablation [1]. Despite this, CTI ablation has remained the preferred option for patients with typical AFL, partly because of the perceived complexity of traditional PVI with RFA, and the high possibility of requiring multiple procedures to achieve durable PVI [1]. As such, in spite of the recognition that CTI ablation often represents only a short-term or partial solution, it continues to be the recommended, first-line treatment approach for symptomatic, recurrent, typical AFL [9, 10].

In recent years, cryoballoon ablation has been developed for AF [11] and has been shown to be non-inferior to RFA in terms of efficacy and safety profile [12]. Cryoballoon ablation is also associated with a shorter learning curve, with more reproducible results across operators and shorter procedure times than RFA, while ensuring a high probability of creating durable PVI [13]. However, the use of cryoballoon PVI only in patients with typical AFL and no prior documentation of AF has not been studied.

## Methods

### Primary endpoint and hypothesis

The primary hypothesis of the CRAFT study is that cryoballoon PVI is superior to CTI ablation as first-line treatment for typical AFL in terms of recurrence of all atrial arrhythmias; it offers the prospect of more complete arrhythmia elimination with a single procedure, while resulting in no increase in procedural risk, or in-catheter laboratory resource utilisation. The primary endpoint is defined as time to first recurrence of sustained (> 30 s) symptomatic atrial arrhythmia, including AF, AFL and atrial tachycardia (AT), following a blanking period of 4 weeks as assessed by implantable loop recorder (ILR) data.

### Secondary endpoints

Key secondary endpoints include (1) time to first episode of AF lasting ≥ 2 min, (2) total AF burden at 12 months, (3) time to recurrence of AFL or AT, (4) procedural and fluoroscopy times and radiation dose and (5) quality of life (QoL) changes at 12 months compared with baseline.

### Safety analysis

The primary safety endpoint is the composite of death, stroke/transient ischaemic attack, cardiac tamponade requiring drainage, atrio-oesophageal fistula, requirement for a permanent pacemaker, serious vascular complications requiring intervention or delaying discharge and persistent phrenic nerve palsy lasting > 24 h. A list of possible procedure-related adverse events is shown in Table 1. A definition of terms is provided in **Supplemental Material**.

**Table 1:** Procedure-related adverse events

Air embolism	
Atrio-oesophageal perforation or fistula	
Atrio-septal defect	
Atrioventricular node damage	
Bleedings events, including groin haematoma	
Cardiac or coronary artery perforation	
Death	
Dysphagia	
Major vascular complication	
Myocardial infarction	
Need for unplanned cardiac or vascular surgery	
Pericardial effusion	
Pericarditis	
Persistent or sustained cardiac arrhythmia	
Phrenic nerve paralysis	
Pulmonary vein stenosis	
Site infection from loop recorder insertion	
Stroke or transient ischaemic attack	

### Study design

CRAFT study is an international, prospective, randomized, open with blinded assessment, multicentre superiority study being performed at 12 sites in the UK and Switzerland. The study is funded by Medtronic Saarl Ltd., but the investigators are solely responsible for the study design and conduct, data collection and analyses, manuscript writing and decision to publish. The study has been approved by the ethical review committees at each site. The trial is being conducted in accordance with the principles of Good Clinical Practice and the Declaration of Helsinki. Block randomisation is used to allocate participants in a 1:1 ratio to either:

- ‘Conventional’ treatment with radiofrequency CTI ablation, OR
- ‘Novel’ treatment with cryoballoon PVI

**Fig. 1:**
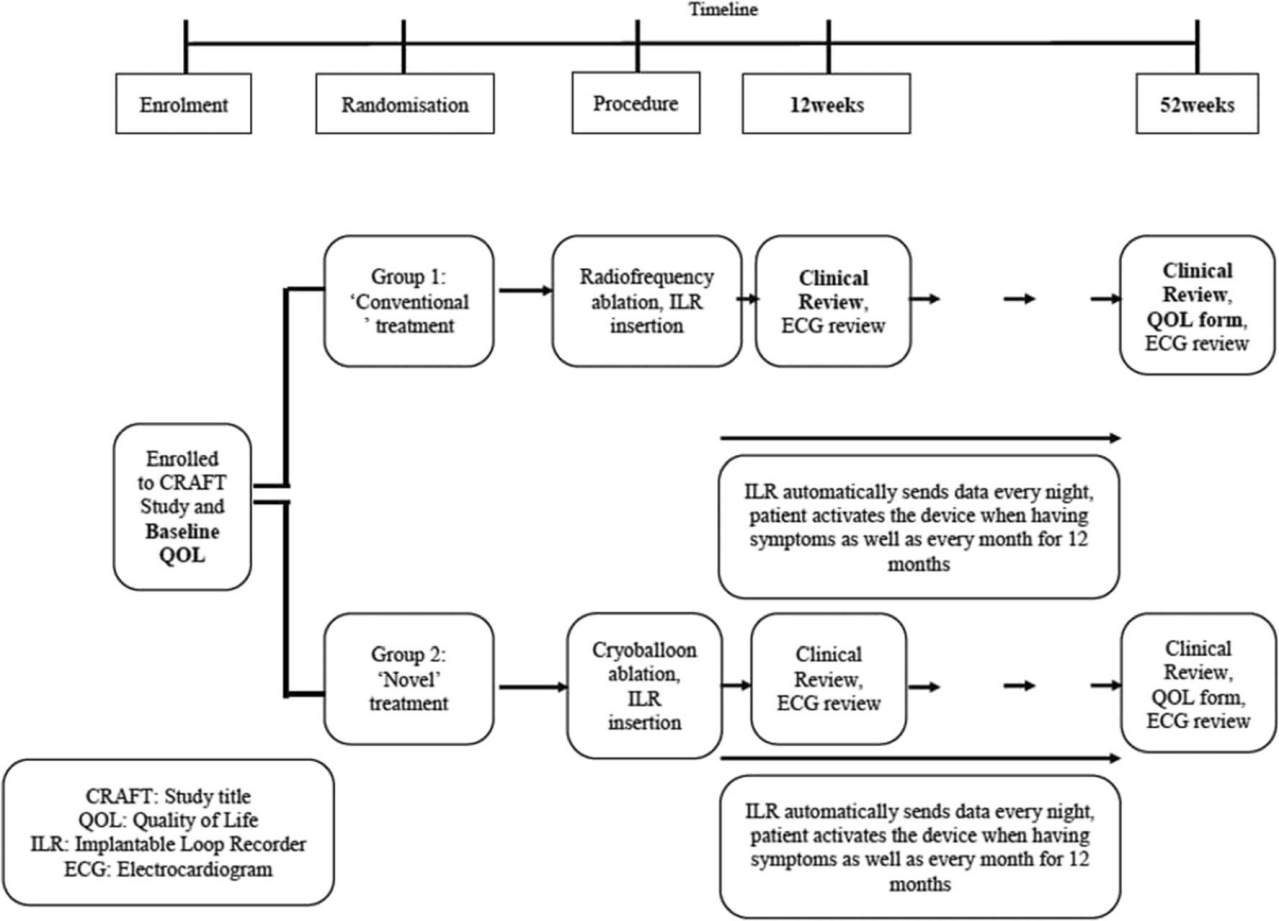
Study flowchart for CRAFT

A study flowchart is included for illustration in Fig. 1.

### Participants

The study population consists of patients with persistent AFL in whom the flutter morphology on standard 12-lead electrocardiogram (ECG) is felt to be compatible with typical, CTI-dependent-AFL. Any documentation of previous AF automatically excludes the patient from the study. At minimum, patients were required to have an ambulatory cardiac monitoring or ECGs recorded on at least 3 different dates to exclude AF. Other key exclusion criteria include flutter diagnosed solely on ambulatory Holter, suspected left atrial AFL, prior CTI ablation or PVI, severe left ventricular systolic dysfunction (left ventricular ejection fraction < 30%) and morbid obesity (body mass index ≥ 40). A full list of exclusion criteria is provided in the **Supplemental Material**. A signed, informed consent form is obtained from every participant prior to randomisation.

### Interventions

Ultrasound-guided vascular access and fluoroscopy guidance are recommended for all cases. For participants presenting to the catheter laboratory in AFL, entrainment is performed from the proximal and distal poles of a catheter situated within the coronary sinus, and CTI to confirm the diagnosis of CTI-dependent AFL. Participants found to have non-CTI-dependent AFL or AF at the time of the procedure will be excluded from undergoing the allocated intervention they have been randomised to. Instead, the investigators will be free to offer them ablation treatment appropriate to their arrhythmia.

#### Radiofrequency CTI ablation

In the control arm, the choice of ablation catheter, settings and overall strategy is at the discretion of individual operators. Using fluoroscopy only, the aim is to achieve bidirectional conduction block across the CTI that persists for at least 15 min following the last radiofrequency application, as has previously been described [14, 15].

#### Cryoballoon PVI

A single trans-septal puncture is performed. Routine administration of heparin is used with a target activated clotting time of > 250. The Arctic Front Advance cryoballoon (Medtronic Inc., Minneapolis, USA) and Achieve Advance Mapping catheter (Medtronic Inc., Minneapolis, USA) are employed for all cases. The aim is to achieve a single 180-s ‘effective’ freeze for each pulmonary vein. A freeze is considered ‘effective’ if either of these criteria is met:

- Time to PVI < 60 s if PV signals are discernible on any pole(s) of the Achieve Advance Mapping catheter, OR
- If PV signals not discernible on any pole(s) of the Achieve Advance Mapping catheter, lowering of balloon temperature below − 40 °C at 60 s

In the event that an effective freeze is not achieved at 60 s, the freeze is terminated, and further ablation attempts are made with a better contrast seal. No ‘bonus’ freeze is routinely applied for any PV once an effective freeze has been delivered. In the case of 2 unsuccessful attempts to achieve an effective freeze for any PV, a 240-s freeze is delivered to the vein, followed by an additional 180-s freeze if PVI is not achieved by the former. Failure to achieve PVI with this maximum of 4 freezes is not pursued with further ablation attempts. Targeted treatment of non-PV triggers or use of focal RF catheter as an adjunct is not allowed by the protocol.

Prior to removal of the left atrial catheters, an assessment of each PV is undertaken with the Achieve Advance Mapping catheter. If spontaneous PV reconnection is identified, cryoballoon application is repeated, where required. Participants in persistent AFL will be cardioverted at the end of the procedure. Protamine administration is allowed prior to sheath removal.

#### Implantable loop recorder

The Reveal LINQ (Medtronic Inc., Minneapolis, USA) is implanted according to the manufacturer’s instructions during the ablation procedure. The programmed algorithm for arrhythmia detection is shown in Table 2.

**Table 2:** Algorithm for programming LINQ devices

Parameter	Settings
Reason for monitoring	Suspected AF
*Detection*	
Tachy	On
Interval rate	146 bpm
Duration	12 beats
Brady	On
Interval rate	30 bpm
Duration	4 beats
Pauses	On
Duration	3 s
AT/AF detection	AF only
Sensitivity	Less sensitive (nominal)
Ectopy rejection	Nominal
AT/AF recording threshold	All episodes

*AF* atrial fibrillation, *AT* atrial tachycardia, *bpm* beats per minute

### Follow-up

The study ‘blanking period’ is defined as the initial 4 weeks following ablation. All AADs are discontinued after this period. Cross-overs are not permitted in the blanking period, with the exception of recurrent AFL with 1:1 atrio-ventricular conduction that is resistant to rate control measures. The use of oral anticoagulation is mandated for a minimum of 2 months and subsequently according to individual stroke risk as assessed by the CHA_2_DS_2_-VASc score. Participants are provided with a symptom diary and asked to transmit monthly ECG recordings from their loop recorder. In addition, participants are instructed to record and transmit ECG recordings following arrhythmia-related symptoms. Transmitted data are reviewed regularly by cardiac physiologists who are blinded to the randomisation status. The follow-up duration is 12 months, with planned study visits at 3 and 12 months, when quality of life assessments are performed with the validated EuroQoL EQ-5D-5L questionnaire. Recurrent atrial arrhythmias are treated at the discretion of the responsible clinician. Clinical events will be adjudicated by a panel of cardiologists, who will be blinded to treatment allocation.

### Sample size calculation

In the PReVENT AF study, incidence of AF following CTI ablation for typical AFL was reported in 52% of participants at 12 months follow-up using an ILR [7]. In a meta-analysis by Maskoun et al., the incidence of AF in a similar patient cohort was 45% over a follow-up period of 16 months [16]. However, this included studies with any form of ambulatory ECG monitoring lasting > 7 days. Based on these data, a 50% incidence of atrial arrhythmias following CTI ablation for typical AFL at 12 months is assumed. We anticipate that cryoballoon PVI will reduce the risk of atrial arrhythmias to 25%. Therefore, with an alpha value of 0.05 and power of 80%, the number of participants required to detect a significant difference for a two-sided test is estimated at 58 per group. To cope with a potential loss to follow-up of 11%, a minimum of 130 subjects will be enrolled in the study.

### Funding and sponsorship

CRAFT is funded by Medtronic International Trading Sarl as part of an Investigator Sponsored Study programme (Grant AF-3908) and is sponsored by Liverpool Heart and Chest Hospital NHS Foundation Trust (UK sites) and University Hospital Basel (Swiss sites).

## Discussion

The CRAFT study is the first randomised controlled trial that compares cryoballoon PVI to radiofrequency CTI ablation for typical AFL. At present, the mode of treatment for symptomatic, recurrent, typical AFL by radiofrequency CTI ablation fails to address the PVs as potential initiating triggers of atrial arrhythmias [4, 5, 17, 18]. Despite evidence to demonstrate better clinical outcomes in terms of arrhythmia-free survival among such patients treated with additional PVI (Table 3), there has been limited uptake with this approach.

**Table 3:** Randomised controlled trials on the role of PVI in isolated AFL

Study	*n*	Ablation strategy	Cardiac monitoring	Follow-up period	Incidence of atrial arrhythmias
Navarette [6]	48	CTI vs CTI + PVI	48-h Holter	16 months	56% vs 13%
PReVENT AF [7]	50	CTI vs CTI + PVI	ILR	12 months	52% vs 12%
REDUCE AF [8]	216	CTI vs CTI + PVI	ILR, event recorder or 7-day Holter	18 months	40% vs 29%
Triple A* [1]	43	CTI vs PVI	ILR	17 months	61% vs 10%

*AF* atrial fibrillation, *AFL* atrial flutter, *CTI* cavo-tricuspid isthmus, *ILR* implantable loop recorder, *PVI* pulmonary vein isolation

*Subset of whole study cohort

Navarrete et al. found that combined CTI ablation and pulmonary vein isolation in patients with typical AFL was associated with a significant improvement in freedom from atrial arrhythmias over a follow-up duration of 16 months compared with CTI ablation alone (56% vs 13%) [6]. Similar findings were reported in PReVENT AF Study I, which was a prospective, single-blind, randomised controlled trial [7]. However, in REDUCE AF, the addition of PVI in patients who underwent CTI ablation for typical AFL only resulted in a significantly lower rate of AF or atrial tachycardia recurrence among those aged > 55 years [8].

The Triple A study has previously demonstrated superiority of standalone PVI over CTI ablation in patients with isolated AFL [1]. However, the limitations of this study include the single-centre study design, small sample size and need for repeat ablation procedures in more than a third of patients in the PVI arm to achieve durable PVI, likely because of the use of RF catheters without contact force measurement capability. However, with the rapidly expanding field of cryoballoon PVI which allows more efficient and durable lesion formation in the PVs, there is a need to evaluate the use of this technology in typical AFL.

## Conclusion

The CRAFT study is an international, multicentre, randomised study comparing clinical outcomes with 2 different approaches to typical AFL—cryoballoon PVI versus radiofrequency CTI ablation. It will increase our understanding of the role of PV triggers in AFL and provide data on whether cryoballoon PVI could be used as an alternative to radiofrequency CTI ablation as a first-line treatment for typical AFL.

## Electronic supplementary material

ESM 1 (DOCX 25 kb)

## Data Availability

Not applicable.

## References

[1] Schneider R, Lauschke J, Tischer T, Schneider C, Voss W, Moehlenkamp F, et al. Pulmonary vein triggers play an important role in the initiation of atrial flutter: initial results from the prospective randomized atrial fibrillation ablation in atrial flutter (triple a) trial. Heart Rhythm. 2015;12:865–71. 10.1016/j.hrthm.2015.01.040. 10.1016/j.hrthm.2015.01.040 25638698

[2] Chinitz JS, Gerstenfeld EP, Marchlinski FE, Callans DJ (2007). Atrial fibrillation is common after ablation of isolated atrial flutter during long-term follow-up. Heart Rhythm.

[3] Celikyurt U, Knecht S, Kuehne M, Reichlin T, Muehl A, Spies F, et al. Incidence of new-onset atrial fibrillation after cavotricuspid isthmus ablation for atrial flutter. Europace. 2017;19(11):1776–80. 10.1093/europace/euw343. 10.1093/europace/euw343 28069839

[4] Waldo AL, Feld GK. Inter-relationships of atrial fibrillation and atrial flutter mechanisms and clinical implications. J Am Coll Cardiol. 2008;51(8):779–86. 10.1016/j.jacc.2007.08.066. 10.1016/j.jacc.2007.08.066 18294560

[5] Waldo AL (2002). Mechanisms of atrial flutter and atrial fibrillation: distinct entities or two sides of a coin?. Cardiovasc Res.

[6] Navarrete A, Conte F, Moran M, Ali I, Milikan N (2011). Ablation of atrial fibrillation at the time of cavotricuspid isthmus ablation in patients with atrial flutter without documented atrial fibrillation derives a better long-term benefit. J Cardiovasc Electrophysiol.

[7] Steinberg JS, Romanov A, Musat D, Preminger M, Bayramova S, Artyomenko S, et al. Prophylactic pulmonary vein isolation during isthmus ablation for atrial flutter: the PReVENT AF study I. Heart Rhythm. 2014;11(9):1567–72. 10.1016/j.hrthm.2014.05.011. 10.1016/j.hrthm.2014.05.011 24832767

[8] Mohanty, S., Natale, A., Mohanty, P., DI Biase, L., Trivedi, C., Santangeli, P., … Dixit, S. (2015). Pulmonary vein isolation to reduce future risk of atrial fibrillation in patients undergoing typical flutter ablation: results from a randomized pilot study (REDUCE AF). J Cardiovasc Electrophysiol, 26(8), 819–825. doi:10.1111/jce.12688. 10.1111/jce.12688 25884325

[9] Blomstrom-Lundqvist C, Scheinman MM, Aliot EM, Alpert JS, Calkins H, Camm AJ (2003). ACC/AHA/ESC guidelines for the management of patients with supraventricular arrhythmias--executive summary: a report of the American College of Cardiology/American Heart Association Task Force on Practice Guidelines and the European Society of Cardiology. J Am Coll Cardiol.

[10] Brugada J, Katritsis DG, Arbelo E, Arribas F, Bax JJ, Blomstrom-Lundqvist C (2019). 2019 ESC Guidelines for the management of patients with supraventricular tachycardia. The task force for the management of patients with supraventricular tachycardia of the European Society of Cardiology (ESC). Eur Heart J.

[11] Packer DL, Kowal RC, Wheelan KR, Irwin JM, Champagne J, Guerra PG (2013). Cryoballoon ablation of pulmonary veins for paroxysmal atrial fibrillation: first results of the North American Arctic Front (STOP AF) pivotal trial. J Am Coll Cardiol.

[12] Kuck K-H, Brugada J, Fürnkranz A, Metzner A, Ouyang F, Chun KRJ, et al. Cryoballoon or radiofrequency ablation for paroxysmal atrial fibrillation. N Engl J Med. 2016;374:2235–45. 10.1056/NEJMoa1602014. 10.1056/NEJMoa1602014 27042964

[13] Reddy VY, Sediva L, Petru J, Skoda J, Chovanec M, Chitovova Z (2015). Durability of pulmonary vein isolation with cryoballoon ablation: results from the sustained pv isolation with Arctic Front Advance (SUPIR) study. J Cardiovasc Electrophysiol.

[14] Shah DC, Takahashi A, Jais P, Hocini M, Clementy J, Haissaguerre M (1999). Local electrogram-based criteria of cavotricuspid isthmus block. J Cardiovasc Electrophysiol.

[15] Vallès E, Cabrera S, Benito B, Alcalde O, Jiménez J, Martí-Almor J (2016). Burning the gap: electrical and anatomical basis of the incremental pacing maneuver for cavotricuspid isthmus block assessment. J Cardiovasc Electrophysiol.

[16] Maskoun W, Pino MI, Ayoub K, Llanos OL, Almomani A, Nairooz R, Hakeem A, Miller J (2016). Incidence of atrial fibrillation after atrial flutter ablation. JACC Clinical electrophysiology.

[17] Haissaguerre M, Jais P, Shah DC, Takahashi A, Hocini M, Quiniou G, et al. Spontaneous initiation of atrial fibrillation by ectopic beats originating in the pulmonary veins. N Engl J Med. 1998;339(10):659–66. 10.1056/NEJM199809033391003. 10.1056/NEJM199809033391003 9725923

[18] Chen SA, Hsieh MH, Tai CT, Tsai CF, Prakash VS, Yu WC, et al. Initiation of atrial fibrillation by ectopic beats originating from the pulmonary veins: electrophysiological characteristics, pharmacological responses, and effects of radiofrequency ablation. Circulation. 1999;100(18):1879–86. 10.1161/01.cir.100.18.1879. 10.1161/01.cir.100.18.1879 10545432

