# Supplementary material for: Cryoballoon pulmonary vein isolation as first line treatment for typical atrial flutter (CRAFT): study protocol for a randomised controlled trial
Source: J Interv Card Electrophysiol. 2020 May 8;60(3):427–32. doi: 10.1007/s10840-020-00746-6 (PMC8134292; doi:10.1007/s10840-020-00746-6)
Supplement: Supplementary file 1 — (DOCX 25 kb) [file 10840_2020_746_MOESM1_ESM.docx]

SUPPLEMENTAL MATERIAL

# Definitions of terms

‘Cardiac tamponade’ is defined as development of pericardial effusion during or within 30 days following ablation, that either causes haemodynamic compromise requiring urgent/elective drainage, or measures ≥1 cm on echocardiography.

‘Persistent phrenic nerve palsy’ is defined as absence of phrenic nerve function with or without function of hemi-diaphragm that does not resolve by the end of the procedure.

‘Serious vascular complication’ includes injury to or occlusion (even transient) of a coronary artery, aortic dissection, femoral pseudo-aneurysm, or arteriovenous fistula, requiring intervention or delaying discharge.

‘Stroke’ is defined as a sudden focal neurologic deficit arising from a presumed cerebrovascular cause rather than from another readily identifiable cause such as a seizure or known tumour, which is not reversible within 24 hours and confirmed on computed tomography (CT) or magnetic resonance imaging (MRI).

‘Transient ischaemic attack’ is a new focal neurologic deficit with rapid symptom resolution (usually within 1-2 hours; always within 24 hours), and neuroimaging with CT/MRI has no evidence of tissue injury.

‘Requirement for a permanent pacemaker’ includes atrio-ventricular nodal damage with partial or complete heart block.

‘Atrio-oesophageal fistula’ is defined as a connection between the atrium and the lumen of the oesophagus documented on CT/MRI.

‘Procedure duration’ is defined as the period from time of femoral puncture to removal of all catheters.

‘Fluoroscopy time’ provides a measure of radiation exposure accounting for all the time spent using fluoroscopy, either calculated or generated automatically by the fluoroscopy system used.

# Exclusion criteria

1. Evidence of previously documented atrial fibrillation (AF)
2. Previous cavo-tricuspid isthmus or AF ablation
3. Atrial flutter (AFL) documented solely on ambulatory monitoring
4. AFL morphology on electrocardiogram suggestive of a left-atrial origin
5. History of AFL with 1:1 atrioventricular conduction and haemodynamic compromise
6. Indwelling atrial septal defect occluder device, or any anatomical reason that precludes access to the left atrium (LA)
7. LA diameter >5.5 cm on parasternal long axis M-mode
8. Severe left ventricular systolic dysfunction (ejection fraction <30% on echocardiography)
9. Stroke or transient ischaemic attack within the last 3 months
10. Inability or unwillingness to undertake oral anticoagulant treatment
11. Morbid obesity (body mass index ≥40)
12. Extreme frailty (score of ≥7 on the Clinical Frailty Scale)
13. Implanted metallic prosthetic valve(s) in mitral position
14. Presence of severe valvular heart disease, as assessed by the responsible cardiologist
15. Indwelling pacemaker, implantable cardioverter-defibrillator or cardiac resynchronisation therapy device
16. Advanced renal dysfunction with an estimated glomerular filtration rate <30 ml/min
17. Currently pregnant
